# Multi-Band Emission of Pr^3+^-Doped Ca_3_Al_2_O_6_ and the Effects of Charge Compensator Ions on Luminescence Properties

**DOI:** 10.3390/nano14010002

**Published:** 2023-12-19

**Authors:** Dejian Hou, Rui Huang, Yi Zhang, Hongliang Li, Wenxing Zhang, Zhisen Lin, Yanqing Guo, Zewen Lin, Jianhong Dong, Jin-Yan Li

**Affiliations:** 1 School of Materials Science and Engineering, Hanshan Normal University, Chaozhou 521041, China; 2 School of Chemical and Environmental Engineering, Hanshan Normal University, Chaozhou 521041, China

**Keywords:** Pr^3+^, multi-band emission, Ca_3_Al_2_O_6_, luminescence

## Abstract

Multi-band emission luminescence materials are of great significance owing to their extensive application in diverse fields. In this research, we successfully prepared a series of Pr^3+^-doped Ca_3_Al_2_O_6_ multi-band emission phosphors via a high-temperature solid-state method. The phase structure, morphology, luminescence spectra and decay curves were investigated in detail. The Ca_3_Al_2_O_6_:Pr^3+^ phosphors can absorb blue lights and emit lights in the 450–750 nm region, and typical emission bands are located at 488 nm (blue), 525–550 nm (green), 611–614 nm (red), 648 nm (red) and 733 nm (deep red). The influence of the Pr^3+^ doping concentration was discussed, and the optimal Pr^3+^ doping concentration was determined. The impacts of charge compensator ions (Li^+^, Na^+^, and K^+^) on the luminescence of Pr^3+^ were also investigated, and it was found that all the charge compensator ions contributed positively to the emission intensity. More importantly, the emission intensity of the as-prepared phosphors at 423 K can still maintain 65–70% of that at room temperature, and the potential application for pc-LED was investigated. The interesting results indicate that the prepared phosphors may serve multifunctional and advanced applications.

## 1. Introduction

In recent years, luminescence materials have attracted great attention owing to their extensive applications, such as daily lighting, backlight displays, biological imaging and medical diagnosis [1,2,3,4,5]. Nowadays, the demand for high-performance materials is growing rapidly with the development of technology. Thus, an investigation on novel luminescence materials is of great significance for both application and basic research. Luminescence center ions play a vital role for phosphors, which determine the luminescence performance of a phosphor to a large extent. Over the past decades, rare earth ions have been widely investigated, as luminescence centers in phosphors, such as Ce^3+^, Eu^3+^, Tb^3+^, Pr^3+^, and Er^3+^ [6,7,8,9,10]. Pr^3+^ is one of the important luminescence activators, which is rich in energy levels. More importantly, the emission spectra of Pr^3+^ in different host lattices can cover ultraviolet to infrared regions [11,12].

Up to now, a large number of Pr^3+^-doped phosphors have been reported. For example, the 5d-4f transition emissions of Pr^3+^ was found to be located in 220–300 nm for the Sr_2_P_2_O_7_:Pr^3+^ phosphor, and continuous ultraviolet-C persistent luminescence could be achieved after X-ray irradiation [13]. Y_3_Si_6_N_11_:Pr^3+^ exhibits a red broad-band emission, which may be potentially used for *w*-LEDs and temperature sensing [14]. Thanks to color-tunable persistent luminescence, the Ca_2_Sb_2_O_7_:Pr^3+^ phosphor is considered to have a bright application prospect in the anti-counterfeiting field [15]. A Ba_3_MgSi_2_O_8_:Pr^3+^ ceramic can change color from white to pink rapidly when irradiated by a UV lamp, and the responses are reversible. These rapid high-contrast reversible properties make Ba_3_MgSi_2_O_8_:Pr^3+^ a prospective material for rewritable paper [16]. Furthermore, the luminescence properties of Pr^3+^-doped LiLuSiO_4_, BaLu_2_Al_2_Ga_2_SiO_12_, CaSc_2_O_4_, BaY_2_Si_3_O_10_, Ca_2_LaTaO_6_, YNbO_4_, etc., have been reported [17,18,19,20,21,22]. Owing to the superior luminescence performance of Pr^3+^-doped materials, Pr^3+^ has been extensively used in lasers, scintillators, *w*-LEDs and optical temperature sensing [23].

The Ca_3_Al_2_O_6_ compound may be a good host matrix due to its excellent physical and chemical stability [24]. To our knowledge, the luminescence of Ce^3+^, Eu^2+^, Dy^3+^, Bi^3+^, Sm^3+^, Tb^3+^, and Mn^2+^ in Ca_3_Al_2_O_6_ has hitherto been investigated [24,25,26,27,28,29]. In this research, considering the advantages of the Ca_3_Al_2_O_6_ host and the Pr^3+^ ion mentioned above, we performed a study on the luminescence properties of Pr^3+^ in the Ca_3_Al_2_O_6_ host. As reported, charge compensator ions may play a vital role to achieve a balance of charges, which could further have great impacts on the luminescence properties of a phosphor [30,31]. Herein, considering the charge imbalance between Ca^2+^ and Pr^3+^, alkali metal R^+^ (R = Li, Na, K) ions were selected as charge compensator ions. The effects of charge compensator ions (Li^+^, Na^+^, and K^+^) were also discussed in detail.

## 2. Materials and Methods

In this research, a series of Pr^3+^-activated multi-band emission phosphors, Ca_3−*x*_Pr*_x_*Al_2_O_6_ and Ca_2.97−*x*_Pr_0.03_R*_x_*Al_2_O_6_ (R^+^ = Li^+^, Na^+^, K^+^), were synthesized through a solid-state reaction technique. The raw starting reactants CaCO_3_ (99.99%, Aladdin, Wallingford, CT, USA), Al_2_O_3_ (99.99%, Aladdin), Pr_2_O_3_ (99.9%, Aladdin), Li_2_CO_3_ (99.9%, Aladdin), Na_2_CO_3_ (99.5%, Aladdin) and K_2_CO_3_ (99.5%, Aladdin) were used for chemical reactions. As is typical for the process of synthesis, the raw reactants were accurately weighed according to the stoichiometric ratio, and then the mixed reactants were ground for about 30 min in an agate mortar. Afterwards, the well-mixed reactants were fully transferred into alumina crucibles and sintered at 1623 K for 4 h in air. Lastly, the samples were cooled down to room temperature naturally and thoroughly ground to obtain fine white powders.

The X-ray diffraction (XRD) patterns were measured using a D8 Advance diffractometer (Cu Kα, λ = 1.5406 Å) for phase analysis. Structure refinements were performed using the TOPAS 5.0-Academic software. Scanning electron micrographs (SEM) were conducted on a field emission scanning electron microscopy (Hitachi SU5000, Tokyo, Japan) for micro-morphology analysis. Diffuse reflectance spectra were measured by a UV3600 spectrofluorometer (Shimadzu, Kyoto, Japan). Luminescence spectra and decay curves at different temperatures were all collected on an Edinburgh FLS1000 spectrofluorometer, and the excitation sources were a 450 W xenon lamp and a μF900 lamp, respectively. The electroluminescence spectra of a pc-LED device were measured on an OHSP-350M LED fast-scan spectrophotometer (Hangzhou Hopoo Light and Color Technology Co., Ltd., Hangzhou, China).

## 3. Results and Discussion

### 3.1. Phase Structure and Morphology

**Figure 1:**
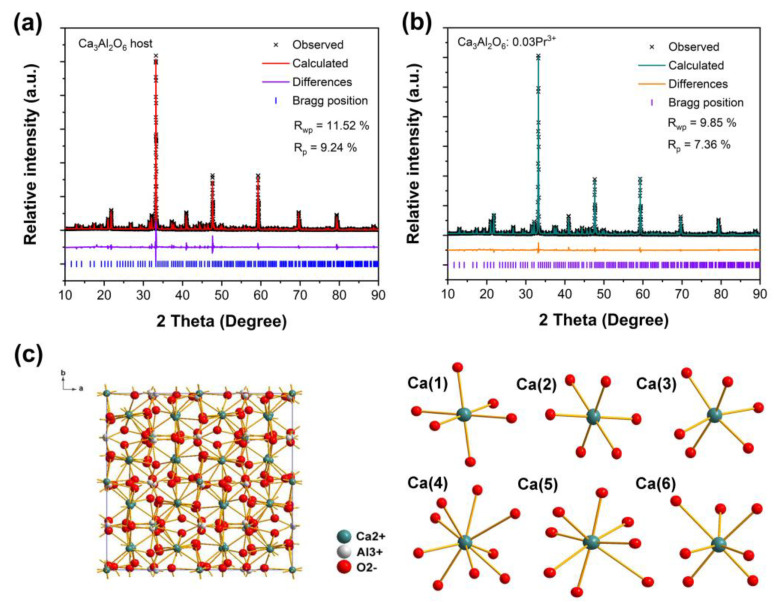
(**a**) X-ray Rietveld refinements of Ca_3_Al_2_O_6_ host; (**b**) X-ray Rietveld refinements of Ca_2.97_Pr_0.03_Al_2_O_6_ phosphor; (**c**) crystal structure of Ca_3_Al_2_O_6_ and the coordination environments of Ca^2+^ in the host.

To confirm the phase structure of the as-prepared phosphors, X-ray Rietveld refinements were performed for two typical samples, Ca_3_Al_2_O_6_ and Ca_2.97_Pr_0.03_Al_2_O_6_. Figure 1a,b depict the refinement results. All the calculated diffraction patterns accord well with the observed ones, which indicates that the as-prepared samples are of a single pure phase. The detailed cell parameters for the X-ray Rietveld refinements are illustrated in Table 1. The undoped and Pr^3+^-doped Ca_3_Al_2_O_6_ phosphors crystallize in a cubic system with a *P*$a\bar{3}$ space group. Due to the similar ionic radii for Ca^2+^ and Pr^3+^ (e.g., r(Ca^2+^) = 1.00 Å, CN = 6; r(Pr^3+^) = 0.99 Å, CN = 6), the cell parameters *a*, *b*, *c*, and cell volumes remain nearly unchanged for Ca_3_Al_2_O_6_ and Ca_2.97_Pr_0.03_Al_2_O_6_. Herein, a small expansion is most probably ascribed to the experimental errors. The crystal structure of the Ca_3_Al_2_O_6_ host is displayed in Figure 1c. The frame structure consists of [AlO_4_] tetrahedrons and [CaO_6_/CaO_7_/CaO_8_/CaO_9_] polyhedrons. There are six different Ca^2+^ sites in this structure. Ca^2+^(1), Ca^2+^(2) and Ca^2+^(3) are coordinated with six oxygen atoms, and the average Ca^2+^-O^2−^ bond lengths are 2.338 Å, 2.391 Å and 2.354 Å, respectively. Ca^2+^(4), Ca^2+^(5), Ca^2+^(6) are coordinated with nine, eight and seven oxygen atoms, and the average Ca^2+^-O^2−^ bond lengths are 2.693 Å, 2.625 Å and 2.525 Å, respectively. The coordination environments of Ca^2+^ are also shown in Figure 1c. In the present case, Pr^3+^ ions may enter all six Ca^2+^ sites, and the luminescence properties we observed should be the whole contribution of Pr^3+^ in Ca^2+^ sites.

**Figure 2:**
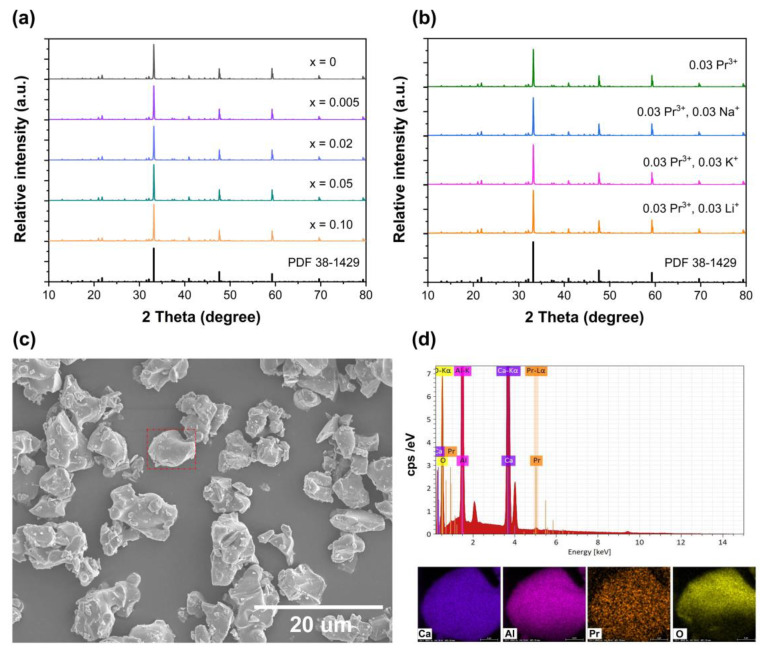
(**a**) XRD patterns of Ca_3−*x*_Pr*_x_*Al_2_O_6_ phosphors; (**b**) XRD patterns of Ca_2.94_Pr_0.03_R_0.03_Al_2_O_6_ (R = Li^+^, Na^+^, K^+^); (**c**) SEM image of Ca_2.97_Pr_0.03_Al_2_O_6_ sample; (**d**) EDS images of one Ca_2.97_Pr_0.03_Al_2_O_6_ particle.

Figure 2a shows the XRD patterns of Pr^3+^-doped phosphors Ca_3−*x*_Pr*_x_*Al_2_O_6_. The observed diffraction peaks are similar in the investigated doping concentration range, and all the diffractions are consistent with the standard card PDF 38-1429[Ca_3_Al_2_O_6_], demonstrating that Pr^3+^ ions were successfully introduced into the Ca_3_Al_2_O_6_ host. The doping of Pr^3+^ did not have a significant impact on the host structure. In addition, a series of Ca_2.97−*x*_Pr_0.03_R*_x_*Al_2_O_6_ (R = Li^+^, Na^+^, K^+^) samples were also prepared, and the XRD results are shown in Figure 2b. As can be seen, the diffraction patterns also accord well with the standard card. All the samples are of a single pure phase. Figure 2c displays the representative SEM image of the Ca_2.97_Pr_0.03_Al_2_O_6_ sample, and the as-prepared sample shows irregular morphology with several micrometers in size. The EDS (energy-dispersive spectroscopy) images were obtained from one particle [marked by a red square] selected from the SEM image in Figure 2c. The elements Ca, Al, Pr and O were successfully detected, as shown in Figure 2d. The elemental mapping results indicate all the elements Ca, Al, O, Pr have been uniformly distributed over the whole particle, and there is no obvious element aggregation in the particles.

### 3.2. Luminescence Properties of Pr^3+^ in Ca_3_Al_2_O_6_

**Figure 3:**
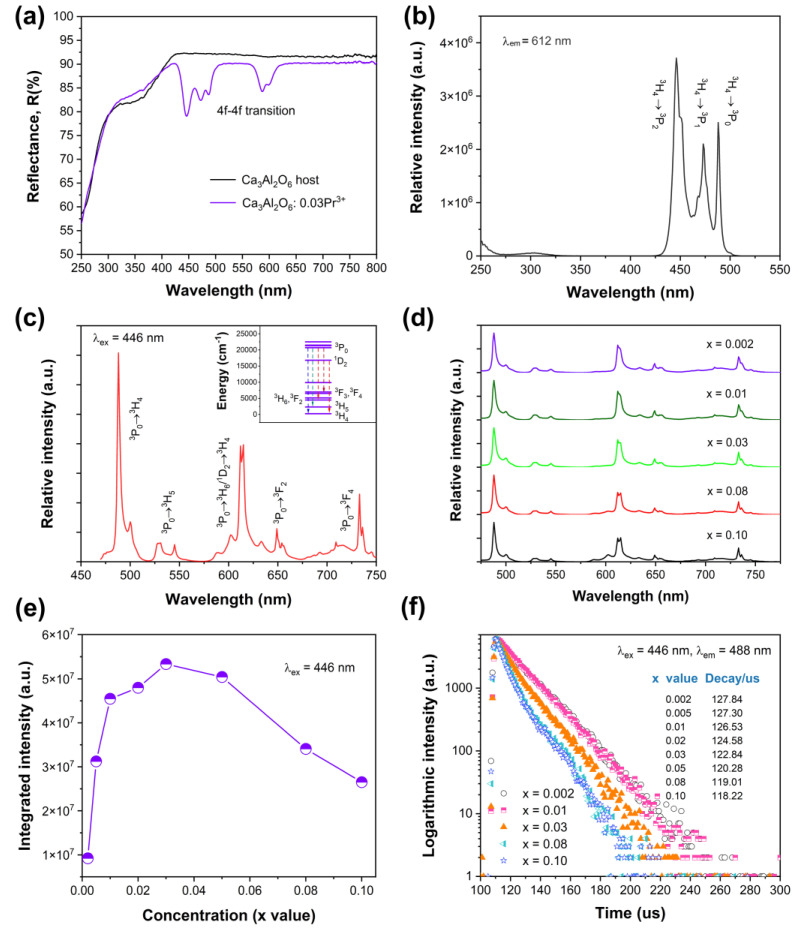
(**a**) Diffuse reflectance spectra of Ca_3_Al_2_O_6_ and Ca_2.97_Pr_0.03_Al_2_O_6_; (**b**) excitation spectrum of Ca_2.97_Pr_0.03_Al_2_O_6_, λ_em_ = 612 nm; (**c**) emission spectrum of Ca_2.97_Pr_0.03_Al_2_O_6_, λ_ex_ = 446 nm; (**d**) emission spectra of Ca_3−*x*_Pr*_x_*Al_2_O_6_ upon 446 nm excitation; (**e**) integrated emission intensity as a function of Pr^3+^ doping concentration upon 446 nm excitation; (**f**) luminescence decay curves of Ca_3−*x*_Pr*_x_*Al_2_O_6_.

Figure 3a illustrates the diffuse reflectance spectra of Ca_3_Al_2_O_6_ and Ca_2.97_Pr_0.03_Al_2_O_6_. A very weak absorption band before 400 nm can be observed, which was ascribed to host-related absorption. The diffuse reflectance spectrum of the Ca_3_Al_2_O_6_ host was in good accordance with the reported one [25]. For the Ca_2.97_Pr_0.03_Al_2_O_6_ sample, a series of sharp absorption lines in the 400–650 nm range also can be observed. In comparison with the pure Ca_3_Al_2_O_6_ host, the absorptions in 400–650 nm are assigned to the 4f-4f transition absorptions of Pr^3+^ in the host. To further characterize the luminescence properties of Ca_2.97_Pr_0.03_Al_2_O_6_, the excitation spectrum and the corresponding emission spectrum are shown in Figure 3b,c, respectively. After monitoring the emission at 612 nm, a series of excitation bands were detected. The sharp excitation bands in the 425–500 nm wavelength range are ascribed to the ^3^H_4_→^3^P_0,1,2_ transition absorptions of Pr^3+^. A very weak band at around 300 nm may relate to the essential absorption of the Ca_3_Al_2_O_6_ host. The excitation spectrum agrees with the diffuse reflectance spectra in Figure 3a. This result indicates that a weak energy transfer from the host lattice to Pr^3+^ may occur. Based on the excitation spectrum, the corresponding emission spectrum was measured, as shown in Figure 3c. Upon 446 nm excitation, a series of sharp emission bands from the blue to deep red region were achieved, which are mainly related to the ^3^P_0_→^3^H_4_, ^3^P_0_→^3^H_5_, ^3^P_0_→^3^H_6_/^1^D_2_→^3^H_4_, ^3^P_0_→^3^F_2_, ^3^P_0_→^3^F_4_ transitions of Pr^3+^ [32,33]. In order to the reveal luminescence process, the energy levels of Pr^3+^ in the 0–25,000 cm^−1^ range are shown in the inset of Figure 3c. Upon 446 nm excitation, the exaction energy was absorbed by Pr^3+^ through ^3^H_4_→^3^P_2_ transitions. Then, electrons returned to ^3^P_0_ and ^1^D_2_ levels via non-radiative relaxation processes, and the emission bands in 450–750 nm were finally observed. Herein, a multi-band emission can be obtained under blue light (446 nm) excitation for samples singly doped with Pr^3+^, which indicates that the phosphor may have potential applications, such as LED application.

When the Pr^3+^ doping concentration increases from 0.002 to 0.10, all the emission spectra are similar [see Figure 3d], demonstrating that the Pr^3+^ doping concentration has little effect on the spectral shape of Ca_3−*x*_Pr*_x_*Al_2_O_6_. However, the integrated emission intensity changes greatly with the doping concentration. As displayed in Figure 3e, the emission intensity greatly increases with increasing Pr^3+^ concentrations at first and reaches a maximum at *x* = 0.03, then it decreases gradually with *x* value owing to the concentration quenching and the non-radiative energy transferring to quenching centers. Normally, the optimal doping concentration is associated with the crucial energy transfer distance (*R_c_*). Herein, the *R_c_* value between Pr^3+^ (activator ions) in the Ca_3_Al_2_O_6_ host could be estimated through the following equation [3]:

(1) $$R_{c}\approx 2{\left(\frac{3V}{4\pi x_{c}N}\right)}^{\frac{1}{3}}$$

where *V* represents the unit cell volume, *N* is the number of Ca^2+^ ions in a unit cell, and *x_c_* refers to the optimal doping concentration. For Pr^3+^-doped Ca_3_Al_2_O_6_, *V* = 3558.45 Å^3^ and *N* = 24. As a consequence, the *R_c_* value is estimated to be 26.63 Å. In general, exchange interaction should be responsible for forbidden transitions with an *R_c_* value less than 5 Å. Clearly, the *R_c_* value is much larger than 5 Å in the present case. Therefore, multipolar interactions should be the dominant factor for the concentration quenching of Pr^3+^.

Figure 3f depicts the luminescence decay curves of ^3^P_0_→^3^H_4_ transition emission (488 nm). The decay processes nearly follow a first-order exponential form at a low Pr^3+^ concentration, and then exhibit certain deviations for a high Pr^3+^ concentration. First, some defects will exist in the phosphors due to the charge imbalance between Ca^2+^ and Pr^3+^, and the high temperature sintering process may also generate some defects as well. The complex defects could affect the excited state relaxation process of Pr^3+^ in the host. Second, the inner energy transfer or interactions between adjacent Pr^3+^ ions increase gradually with the Pr^3+^ doping concentration. Third, the multi-site luminescence of Pr^3+^ exists in Ca_3_Al_2_O_6_, and the luminescence decay for Pr^3+^ in each Ca^2+^ site may also show some differences. Therefore, the decay curves of Pr^3+^ exhibit bi-exponential or even multi-exponential decay behaviors with increasing the Pr^3+^ doping concentration. Because of the deviations, the average decay constants can be estimated using Equation (2) [34]:

(2) $$\tau =\frac{\int_{0}^{\infty }I(t)tdt}{\int_{0}^{\infty }I(t)dt}$$

The estimated decay constants are also shown in Figure 3f. The decay constants shortened from 127.84 μs (*x* = 0.002) to 118.22 μs (*x* = 0.10). For luminescence materials singly doped with Pr^3+^, the average lifetime *τ* is the reciprocal sum of all the radiative transition (*W_R_*) and non-radiative transition (*W_NR_*), as can be described by Equation (3) [35]:

(3) $$\tau =\frac{1}{w_{R}+w_{NR}}$$

**Figure 4:**
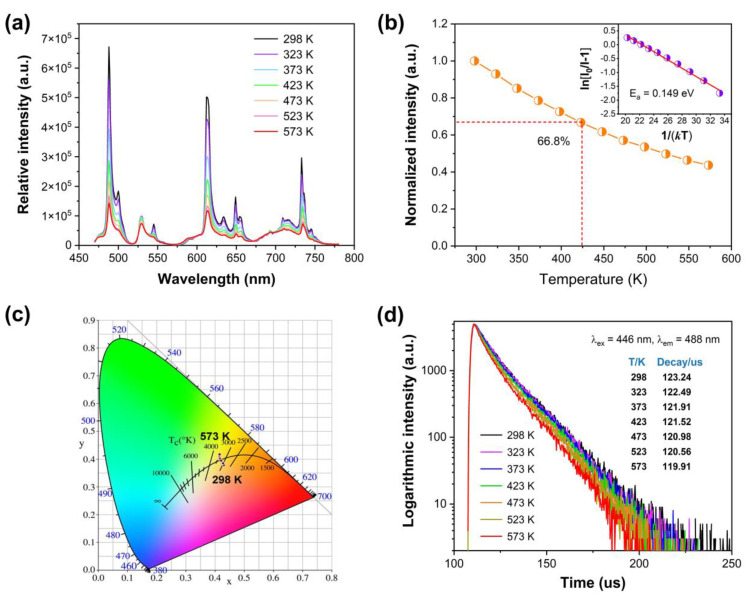
(**a**) Emission spectra of Ca_2.97_Pr_0.03_Al_2_O_6_ at various temperatures; (**b**) normalized emission intensity dependent on temperature, and inset displays the relationship between ln[(*I*_0_/*I*) − 1] and 1/(*kT*); (**c**) CIE chromaticity coordinates of Ca_2.97_Pr_0.03_Al_2_O_6_ at different temperatures; (**d**) luminescence decay curves of Ca_2.97_Pr_0.03_Al_2_O_6_ at different temperatures.

Herein, the decrease in *τ* confirms the increasing non-radiative energy transfer with *x* value. The influence of temperature is a key factor for further applications. Figure 4a shows the emission spectra of Ca_2.97_Pr_0.03_Al_2_O_6_ at various temperatures. The ^3^P_0_→^3^H_4_, ^3^P_0_→^3^H_5_, ^3^P_0_→^3^H_6_/^1^D_2_→^3^H_4_, ^3^P_0_→^3^F_2_, ^3^P_0_→^3^F_4_ transition emission lines of Pr^3+^ can be observed, All the emission spectra are similar, but the emission intensity changes remarkably. Figure 4b displays the emission intensity dependent on temperature. The relative emission intensity decreases gradually with increasing temperature owing to the temperature-involved thermal quenching. The emission intensity at 423 K maintains about 66.8% of that at 298 K (room temperature). In general, the Δ*E_a_* value (activation energy) can be used to evaluate the thermal quenching properties of a phosphor, and the relevant equation is described as following [36]:

(4) $$I_{T}=\frac{I_{0}}{1+A*exp(-{\Delta E}_{a}/kT)}$$

where *I*_0_ and *I_T_* refer to the initial emission intensity and the intensity at a given temperature *T*, respectively. *k* represents the Boltzmann constant, and *A* can be treated as constant in specific cases. The Equation (4) could also be expressed as [37]

(5) $$\ln\left(\frac{I_{0}}{I}-1\right)=lnA-\frac{\Delta E_{a}}{kT}$$

As a consequence, the activation energy Δ*E_a_* can be obtained according to the relationship between ln[(*I*_0_/*I*) − 1] and 1/(*kT*). As depicted in the inset of Figure 4b, a well-fitted straight line with a slope of −0.149 was achieved. Thus, the Δ*E_a_* value is 0.149 eV for Ca_2.97_Pr_0.03_Al_2_O_6_. As reported, the Δ*E_a_* value for the Ca_2_ZnSi_2_O_7_:0.005Pr^3+^ phosphor is 0.2255 eV [38], and the values are 0.22 eV, 0.18 eV for Pr^3+^-doped SrLaMgTaO_6_:Pr^3+^ and BaLaMgTaO_6_:Pr^3+^, respectively [35]. The Δ*E_a_* value in this research is similar to that of Ca_9_MgLi(PO_4_)_7_:Pr^3+^ (Δ*E_a_* = 0.15 eV), which is slightly larger than that of CaLaB_7_O_13_:Pr^3+^ (Δ*E_a_* = 0.116 eV) [39,40].

Figure 4c shows the CIE (Commission International de I′Eclairage 1931) chromaticity coordinates for the emission of Ca_2.97_Pr_0.03_Al_2_O_6_ at various temperatures. Although all the emission lines can be observed in Figure 4a, the chromaticity coordinates also show some differences, which move from (0.431, 0.379) at 298 K to (0.416, 0.417) at 573 K due to the thermal population of electrons between the ^3^P_0_ and ^1^D_2_ levels. The emission colors are located at the orange–yellow region in all the temperature ranges.

Upon 446 nm excitation and detecting the emission at 488 nm, temperature-dependent luminescence decay curves were collected and illustrated in Figure 4d. The luminescence decay times become shorter and shorter with increasing temperature, which also demonstrates the increasing non-radiative energy transfer processes. These results are consistent with the temperature-dependent emission spectra in Figure 4a.

### 3.3. The Influences of Charge Compensator Ions

**Figure 5:**
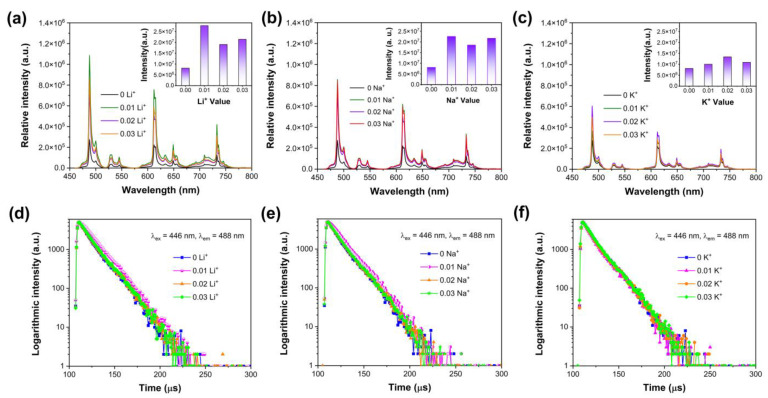
(**a**) Emission spectra of Ca_2.97−*x*_Pr_0.03_Li*_x_*Al_2_O_6_ (*x* = 0, 0.01, 0.02, 0.03) under 446 nm excitation; (**b**) emission spectra of Ca_2.97−*x*_Pr_0.03_Na*_x_*Al_2_O_6_ (*x* = 0, 0.01, 0.02, 0.03) under 446 nm excitation; (**c**) emission spectra of Ca_2.97−*x*_Pr_0.03_K*_x_*Al_2_O_6_ (*x* = 0, 0.01, 0.02, 0.03) under 446 nm excitation; (**d**) luminescence decay curves of Ca_2.97−*x*_Pr_0.03_Li*_x_*Al_2_O_6_ (*x* = 0, 0.01, 0.02, 0.03) at room temperature; (**e**) luminescence decay curves of Ca_2.97−*x*_Pr_0.03_Na*_x_*Al_2_O_6_ (*x* = 0, 0.01, 0.02, 0.03) at room temperature; (**f**) luminescence decay curves of Ca_2.97−*x*_Pr_0.03_K*_x_*Al_2_O_6_ (*x* = 0, 0.01, 0.02, 0.03) at room temperature.

In the above section, the phosphors were designed by nonequivalent substitution, that is, one Pr^3+^ substitutes one Ca^2+^ in the Ca_3_Al_2_O_6_ host. Therefore, charge defects will exist due to the nonequivalent substitution, which may have impacts on the luminescence of Pr^3+^. Figure 5a shows the emission spectra of Ca_2.97−*x*_Pr_0.03_Li*_x_*Al_2_O_6_ (*x* = 0, 0.01, 0.02, 0.03) samples. The introduction of compensator ions Li^+^ does not significantly influence the emission spectral shape. The inset depicts the integrated emission intensity at various Li^+^ concentrations. The co-doping of Li^+^ contributes positively to the emission intensity of Pr^3+^. The emission intensity is about 3.4 (0.01 Li^+^), 2.3 (0.02 Li^+^), 2.6 (0.03 Li^+^) times that of Ca_2.97_Pr_0.03_Al_2_O_6_, respectively. The emission spectra of Ca_2.97−*x*_Pr_0.03_Na*_x_*Al_2_O_6_ and Ca_2.97−*x*_Pr_0.03_K*_x_*Al_2_O_6_ samples are displayed in Figure 5b,c. The incorporation of Na^+^ and K^+^ can also improve the emission intensity of Pr^3+^. The relative emission intensity is about 2.8 (0.01 Na^+^), 2.3 (0.02 Na^+^), 2.7 (0.03 Na^+^), 1.2 (0.01 K^+^), 1.6 (0.02 K^+^), 1.3 (0.03 K^+^) times that of Ca_2.97_Pr_0.03_Al_2_O_6_, respectively. Among all the samples, the optimal emission intensity can be achieved for Ca_2.96_Pr_0.03_Li_0.01_Al_2_O_6_. For the Ca_2.97_Pr_0.03_Al_2_O_6_ sample, defects may be caused via several paths [31,41,42]: (1) Three Ca^2+^ replaced by two Pr^3+^ ions generates a Ca^2+^ vacancy at the same time, which can be described by 3Ca_Ca_→2${Pr}_{Ca}^{\cdot }$+$V_{Ca}^{\prime \prime }$. (2) Two Ca^2+^ ions replaced by two Pr^3+^ ions may cause an interstitial $O_{i}^{\prime \prime }$ defect. (3) Two Ca^2+^ ions replaced by two Pr^3+^ ions may cause an oxygen vacancy $V_{O}^{\prime \prime }$ according to the possible process 2Ca_Ca_→2${Pr}_{Ca}^{\cdot }$+$V_{O}^{\prime \prime }$. In fact, some defects could act as killers of luminescence centers, resulting in the quenching of luminescence intensity. For Ca_2.97−*x*_Pr_0.03_R*_x_*Al_2_O_6_ samples, two Ca^2+^ ions would be substituted by one Pr^3+^ ion and one R^+^ ion according to 2Ca_Ca_→${Pr}_{Ca}^{\cdot }$+$R_{Ca}^{\prime }$. Some vacancies or defects were reduced. Therefore, the observed luminescence intensity can be improved. Furthermore, the ionic radii of K^+^ and Na^+^ are larger than that of Li^+^. Li^+^ ions more easily fill the vacancy defects, which may also further promote the effective entrance of the Pr^3+^ into Ca^2+^ sites in the host [31,43,44]. As a consequence, the emission intensity can be significantly enhanced by the introduction of Li^+^ ions into the host lattice. The influence of charge compensator ions on some phosphors have been reported, such as BaZrGe_3_O_9_:Cr^3+^, Ca_2_GdTaO_6_:Mn^4+^, M (M = Li^+^, Na^+^, K^+^, and Mg^2+^), Ca_2_ZnSi_2_O_7_:Pr^3+^ and α-Sr_2_P_2_O_7_:Dy^3+^ [38,42,45,46].

Luminescence decay curves of Ca_2.97−*x*_Pr_0.03_Li*_x_*Al_2_O_6_, Ca_2.97−*x*_Pr_0.03_Na*_x_*Al_2_O_6_ and Ca_2.97−*x*_Pr_0.03_K*_x_*Al_2_O_6_ were collected at room temperature to confirm the influence of charge compensator ions, as illustrated in Figure 5d–f. As can be observed, several luminescence decay curves show notable increase in comparison with Ca_2.97_Pr_0.03_Al_2_O_6_, especially for Ca_2.96_Pr_0.03_Li_0.01_Al_2_O_6_ and Ca_2.96_Pr_0.03_Na_0.01_Al_2_O_6_. When charge compensator ions were introduced into the host lattice, the defects and interactions between adjacent Pr^3+^ ions were be changed. Luminescence decay curves further demonstrate that non-radiative energy transfer processes have been reduced, which leads to the increases of emission intensity in Figure 5a–c.

**Figure 6:**
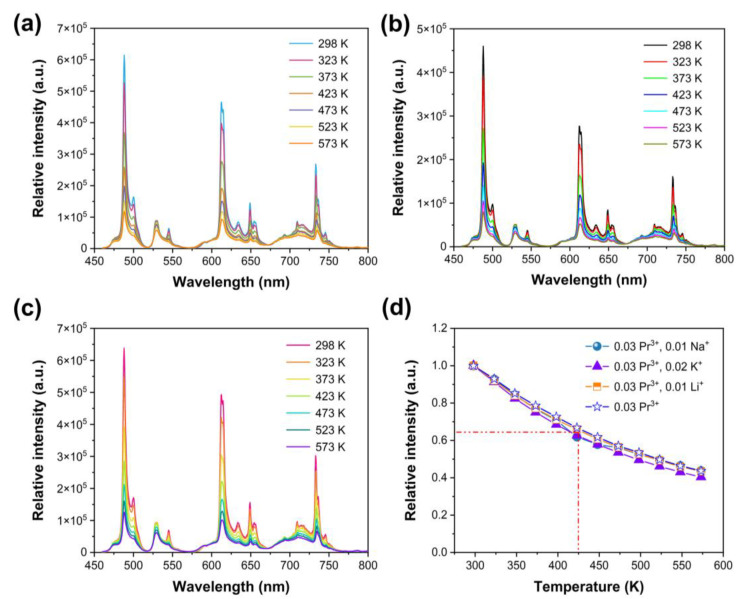
(**a**) Temperature-dependent emission spectra of Ca_2.96_Pr_0.03_Li_0.01_Al_2_O_6_ under 446 nm excitation; (**b**) temperature-dependent emission spectra of Ca_2.96_Pr_0.03_Na_0.01_Al_2_O_6_ upon 446 nm excitation; (**c**) temperature-dependent emission spectra of Ca_2.95_Pr_0.03_K_0.02_Al_2_O_6_ under 446 nm excitation; (**d**) normalized emission intensity dependent of different temperatures.

To evaluate the influence of charge compensator ions on thermal quenching properties, temperature dependent emission spectra and luminescence decay curves were measured for Ca_2.96_Pr_0.03_Li_0.01_Al_2_O_6_, Ca_2.96_Pr_0.03_Na_0.01_Al_2_O_6_ and Ca_2.95_Pr_0.03_K_0.02_Al_2_O_6_. Emission intensity declines with increasing temperature for Li^+^, Na^+^ and K^+^ co-doped samples, and all the emission profiles are similar as shown in Figure 6a–c. Normalized integrated emission intensity dependent on different temperatures are listed in Figure 6d. The observed emission intensities at 423 K are all about 65–70% of those at 298 K, which are similar to that of Ca_2.97_Pr_0.03_Al_2_O_6_.

**Figure 7:**
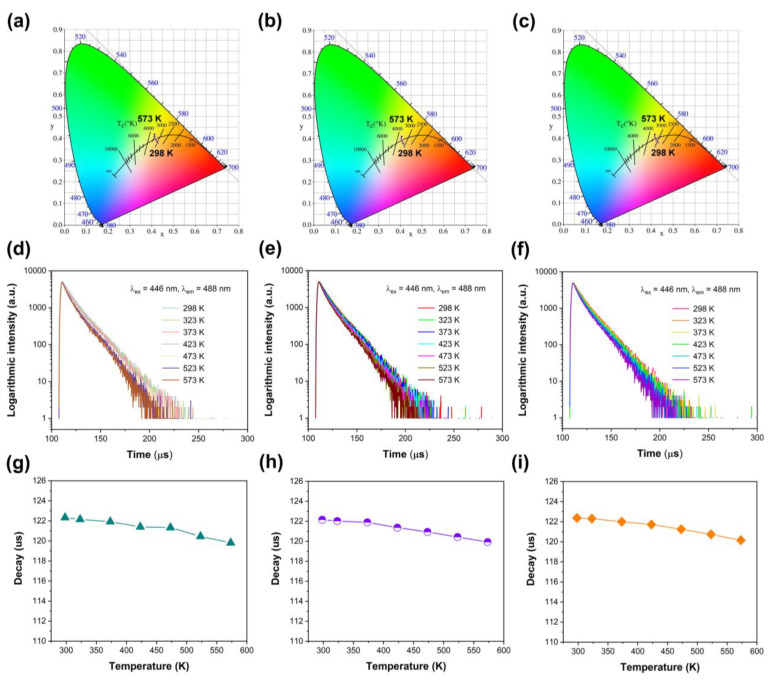
(**a**) CIE chromaticity coordinates of Ca_2.96_Pr_0.03_Li_0.01_Al_2_O_6_ at different temperatures; (**b**) CIE chromaticity coordinates of Ca_2.96_Pr_0.03_Na_0.01_Al_2_O_6_ at different temperatures; (**c**) CIE chromaticity coordinates of Ca_2.95_Pr_0.03_K_0.02_Al_2_O_6_ at different temperatures; (**d**) luminescence decay curves of Ca_2.96_Pr_0.03_Li_0.01_Al_2_O_6_ at different temperatures; (**e**) luminescence decay curves of Ca_2.96_Pr_0.03_Na_0.01_Al_2_O_6_ at different temperatures; (**f**) luminescence decay curves of Ca_2.95_Pr_0.03_K_0.02_Al_2_O_6_ at different temperatures; (**g**) luminescence decay times of Ca_2.96_Pr_0.03_Li_0.01_Al_2_O_6_ dependent on temperature; (**h**) luminescence decay times of Ca_2.96_Pr_0.03_Na_0.01_Al_2_O_6_ dependent on temperature; (**i**) luminescence decay times of Ca_2.95_Pr_0.03_K_0.02_Al_2_O_6_ dependent on temperature.

The CIE chromaticity coordinates of Ca_2.96_Pr_0.03_Li_0.01_Al_2_O_6_, Ca_2.96_Pr_0.03_Na_0.01_Al_2_O_6_ and Ca_2.95_Pr_0.03_K_0.02_Al_2_O_6_ at different temperatures are shown in Figure 7a–c. The variation tendencies are the same and accord with the Ca_2.97_Pr_0.03_Al_2_O_6_ sample in Figure 4c. Luminescence decay curves and decay times at various temperatures are illustrated in Figure 7d–i. Luminescence decay processes become faster and gradually deviate from the first-order exponential, owing to the heat-involved energy transfer. The average decay times were also estimated by Equation (2), and the results are shown in Figure 7g–i. The decay times decrease from 122.37 μs (298 K) to 120.16 μs (573 K) for Ca_2.96_Pr_0.03_Li_0.01_Al_2_O_6_. The values are 122.33 μs (298 K, Na^+^ doped), 119.81 μs (573 K, Na^+^ doped), 122.16 us (298 K, K^+^ doped), 119.93 μs (573 K, K^+^ doped). The decreases in decay times are also similar for the three samples, which are very consistent with the observed temperature-dependent emission spectra. Based on the above discussions, it is can be found that certain amounts of compensator ions will enhance the emission intensity of Pr^3+^, especially for Li^+^ co-doped ones.

### 3.4. Potential Applications

**Figure 8:**
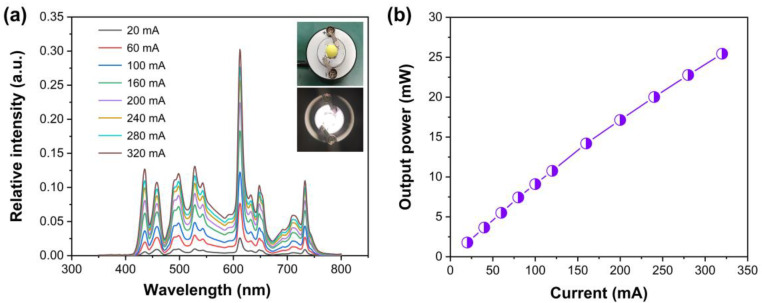
(**a**) Emission spectra of pc-LED device driven at 20–320 mA, and inset shows the pictures of pc-LED prototype and working LED; (**b**) output optical power on dependent of driven current.

A phosphor-converted light-emitting diode (pc-LED) was fabricated with a blue LED chip, Y_3_Al_5−x_Ga_x_O_12_:Ce^3+^ (YAGG:Ce, yellow–green component) and Ca_2.96_Pr_0.03_Li_0.01_Al_2_O_6_. Multi-emission bands from ~425 nm to 750 nm were detected at 20–320 mA driven currents, as shown in Figure 8a. Herein, differences appear in comparisons with the above emission spectra. Several factors may contribute to this: ① The responses of the relative emission intensity some exhibit are different for different spectrophotometers. ② The filters used in measurement setup. ③ Most importantly, the absorption of YAGG:Ce phosphor in blue region. The emission intensity of the pc-LED device increases gradually with driven currents. We did not observe light saturation in the 20–320 mA current range. The inset of Figure 8a displays the photographs of the pc-LED, and bright white light can be observed clearly with the driven power on. CIE chromaticity coordinates of the working pc-LED are (0.3682, 0.3598), and the CRI (color rendering index) and CCT (correlated color temperature) are 81.9 and 4236 K driven by 160 mA current, respectively. The output optical power also increases with a driven current, as shown in Figure 8b. The luminous efficiency in this situation is around 8.99 lm/W, and the maximum photoelectric efficiency is about 3.5%.

## 4. Conclusions

In summary, a series of Pr^3+^-doped Ca_3_Al_2_O_6_ with multi-band emission were successfully designed and prepared. All the samples crystallize in cubic system, but the emission intensity is strongly dependent on the Pr^3+^ doping concentration. The optimal Pr^3+^ concentration is 0.03, and the crucial energy transfer distance *R_c_* was determined to be 26.63 Å. Pr^3+^-doped phosphors exhibit good thermal quenching properties. The emission intensity at 423 K can maintain 65–70% of that at room temperature, and the estimated activation energy Δ*E_a_* is 0.149 eV for Ca_2.97_Pr_0.03_Al_2_O_6_. The introduction of charge compensator ions can greatly enhance the emission intensity of Pr^3+^ due to a possible decrease in charge defects, especially for the Li^+^ co-doped ones. The luminescence intensity of Ca_2.96_Pr_0.03_Li_0.01_Al_2_O_6_ can be increased by 340% in comparison to that of Ca_2.97_Pr_0.03_Al_2_O_6_. A white light emission pc-LED was created using Y_3_Al_5−x_Ga_x_O_12_:Ce^3+^ and Ca_2.96_Pr_0.03_Li_0.01_Al_2_O_6_ as color converters. The CIE coordinates of the working pc-LED are (0.3682, 0.3598), and the CRI and CCT are 81.9 and 4236 K under 160 mA current. Thanks to good multi-band emission properties, the designed phosphors may have potential applications.

## Figures and Tables

**Table 1:** Refined cell parameters of Ca_3_Al_2_O_6_ and Ca_2.97_Pr_0.03_Al_2_O_6_.

Samples	Ca_3_Al_2_O_6_ Host	Ca_2.97_Pr_0.03_Al_2_O_6_
Space group	*P* $a\bar{3}$	*P* $a\bar{3}$
*a* = *b* = *c* (Å)	15.26326 (6)	15.26700 (4)
*α* = *β* = *γ* (°)	90	90
Cell volume (Å^3^)	3555.86 (4)	3558.45 (5)
*R_p_* (%)	9.24	7.36
*R_wp_* (%)	11.52	9.85

## Data Availability

The data underlying the results presented in this paper are not publicly available at this time but may be obtained from the authors upon reasonable request.
